# SARS‐CoV‐2 infection‐induced immunity and the duration of viral shedding: Results from a Nicaraguan household cohort study

**DOI:** 10.1111/irv.13074

**Published:** 2022-12-01

**Authors:** Hannah E. Maier, Miguel Plazaola, Roger Lopez, Nery Sanchez, Saira Saborio, Sergio Ojeda, Carlos Barilla, Guillermina Kuan, Angel Balmaseda, Aubree Gordon

**Affiliations:** ^1^ Department of Epidemiology, School of Public Health University of Michigan Ann Arbor Michigan USA; ^2^ Sustainable Sciences Institute Managua Nicaragua; ^3^ Centro Nacional de Diagnóstico y Referencia Ministry of Health Managua Nicaragua; ^4^ Centro de Salud Sócrates Flores Vivas Ministry of Health Managua Nicaragua

**Keywords:** COVID‐19, immunity, reinfection, SARS‐CoV‐2, viral shedding

## Abstract

**Background:**

Much of the world's population has been infected with SARS‐CoV‐2. Thus, immunity from prior infection will play a critical role in future SARS‐CoV‐2 transmission. We investigated the impact of infection‐induced immunity on viral shedding duration and viral load.

**Methods:**

We conducted a household cohort study in Managua, Nicaragua, with an embedded transmission study that closely monitors participants regardless of symptoms. Real‐time reverse‐transcription polymerase chain reaction (RT‐PCR) and enzyme‐linked immunosorbent assays (ELISAs) were used to measure infections and seropositivity, respectively. Blood samples were collected twice annually and surrounding household intensive monitoring periods. We used accelerated failure time models to compare shedding times. Participants vaccinated ≥14 days prior to infection were excluded from primary analyses.

**Results:**

There were 600 RT‐PCR‐confirmed SARS‐CoV‐2 infections in unvaccinated participants between May 1, 2020, and March 10, 2022, with prior ELISA data. Prior infection was associated with 48% shorter shedding times (event time ratio [ETR] 0.52, 95% CI: 0.39–0.69, mean shedding: 13.7 vs. 26.4 days). A fourfold higher anti‐SARS‐CoV‐2 spike titer was associated with 17% shorter shedding (ETR 0.83, 95% CI: 0.78–0.90). Similarly, maximum viral loads (lowest cycle threshold [CT]) were lower for previously infected individuals (mean CT 29.8 vs. 28.0, *p* = 4.02 × 10^−3^), for adults and children ≥10 years, but not for children 0–9 years; there was little difference in CT levels for previously infected versus naïve adults aged above 60 years.

**Conclusions:**

Prior infection‐induced immunity was associated with shorter viral shedding and lower viral loads, which may be important in the transition from pandemic to endemicity.

## 1 INTRODUCTION

As the SARS‐CoV‐2 pandemic continues into its third year, over 40% of the world's population has been infected,^1^ making it critical to understand how immunity from prior infection affects repeat infection and transmission, particularly for low‐ and middle‐income countries where vaccination rates are lower.^2^

We used data from an existing household cohort study of 2539 individuals 0 to 94 years of age in Managua, Nicaragua.^3^ We compare SARS‐CoV‐2 viral shedding duration and viral load between previously infected and serologically naïve individuals, as well as by antibody levels.

## 2 METHODS

### 2.1 The household influenza cohort study (HICS)

**FIGURE 1:**
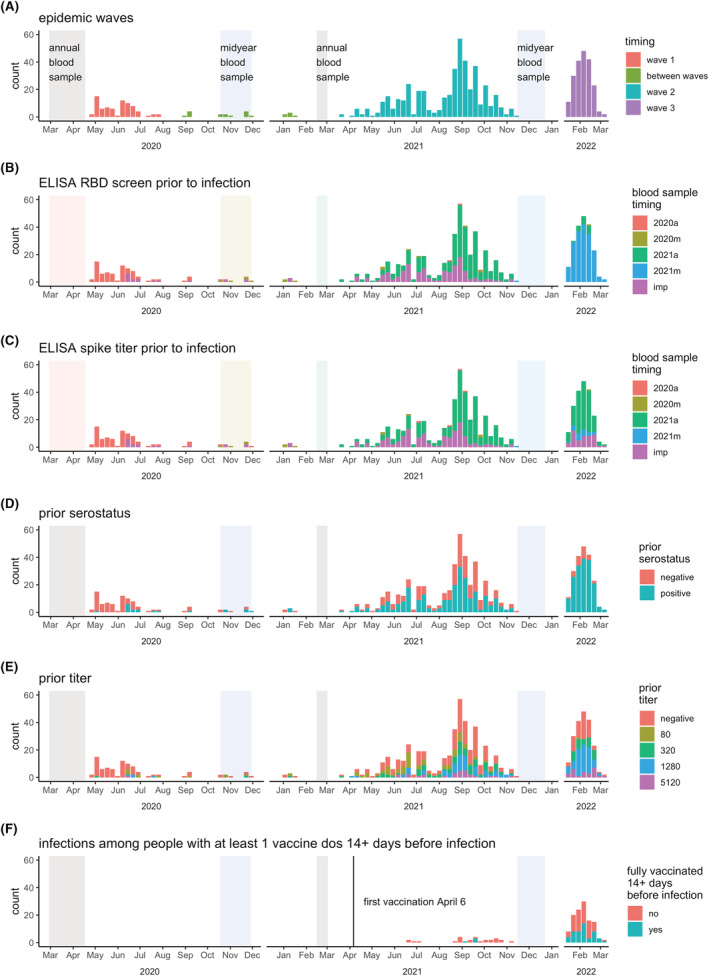
Epidemic timing. Weekly RT‐PCR counts are colored according to (A) epidemic wave, (B–C) most recent blood sample for ELISA RBD screen and spike titer, (D–E) RBD screen and spike titer results, and F) full vaccination status. Panel (F) is a subset to show only infections in people with ≥1 vaccination ≥14 days prior to infection. IMP, intensive monitoring period. In (B) and (C), annual and midyear blood sampling periods are colored to match the legend so the timing of prior blood samples associated with infections can be easily identified; it can be seen here that many 2022 infections have blood samples for titers from the annual 2021 sample.

HICS is an ongoing prospective cohort study of influenza in households that are free of disease at baseline (Figure S1). Located in district II of Managua, Nicaragua, HICS began in 2017 and was expanded in February 2020 to examine SARS‐CoV‐2 infection and disease. At the first indication of any illness, participants are requested to report to the study health center, where they are provided with their primary care. A transmission study is nested within HICS (Figure S2), in which participants are monitored closely and tested regardless of symptoms once a SARS‐CoV‐2 case is detected in their household allowing for the detection of cases regardless of symptoms. Once households are activated, study staff visit regularly, at approximately days 0, 3, 7, 14, 21, and 30, to collect combined nasal/oropharyngeal swabs and symptom diaries. Blood samples for serology were collected annually in March–April (annual samples) and October–December (midyear samples) and surrounding intensive monitoring periods (IMPs; at household activation and 30–45 days later; Figure 1A–C).

This study was approved by the institutional review boards at the Nicaraguan Ministry of Health and the University of Michigan (HUM00119145 and HUM00178355). Informed consent or parental permission was obtained for all participants. Assent was obtained from children aged ≥6 years.

### 2.2 Laboratory assays

Real‐time reverse‐transcription polymerase chain reaction (RT‐PCR) was performed according to the protocol from Chu et al.^4^ Enzyme‐linked immunosorbent assays (ELISAs) were run on paired serum samples (current vs. baseline) with a protocol adapted from the Krammer laboratory.^5^ The SARS‐CoV‐2 spike receptor binding domain (RBD) and spike proteins for ELISAs were produced in single batches at the Life Sciences Institute at the University of Michigan; these were generated based on the original SARS‐CoV‐2 strain. RBD was used for screening (positive/negative) as it is more specific than spike, and spike was used to titer samples that screened positive by RBD ELISA. The limits of detection for endpoint titers were 100 (lower) and 6400 (upper). All RT‐PCR and most ELISAs were performed at the Nicaraguan National Virology Laboratory, with a minority of 2020 annual samples processed at the University of Michigan. Sequencing information was described previously.^6^

### 2.3 Viral shedding

RT‐PCR‐positive episodes were considered separate episodes if they were ≥60 days apart.

Viral shedding durations (Figure S3) were defined as either (1) equal to or greater than the number of days detected RT‐PCR‐positive (right censored) or (2) between the number of days RT‐PCR‐positive and the time between prior and subsequent negative RT‐PCR tests (interval censored).

### 2.4 Vaccination

To assess seropositivity resulting from prior infection, individuals with any vaccine dose ≥14 days prior to shedding onset were excluded for primary analyses. Additionally, an analysis was run to compare full vaccination to otherwise seropositive (could have incomplete vaccination) and seronegative.

### 2.5 Analysis

Participant age was calculated at the time of infection. Antibody titers were log‐transformed and rounded for all analyses (log4[titer/5]) to reflect serial dilutions (original titer values of 5, 80, 320, 1280, and 5120 were analyzed as 0, 1, 2, 3, 4, and 5). Detectable spike titers without an RBD screen (e.g., when participants were previously RBD‐positive) were coded as seropositive, and negative RBD‐screened samples without a spike titer (e.g., titers not available for most 2021 midyear samples) were coded as a non‐detectable spike titer. Because respiratory samples were not collected continuously, but at intervals (Figure S3), the precise duration of viral shedding could not be known, but models can account for this uncertainty. Viral shedding durations were classified as “right censored” if either the first or last respiratory sample was RT‐PCR positive, so that only the minimum shedding duration was known, or they were classified as “interval censored” if a positive RT‐PCR was observed between two negative RT‐PCRs, so that the minimum and maximum days of shedding were known (Figure S3). Accelerated failure time (AFT) models, which can handle censored outcomes, were used to compare shedding durations by prior immunity, using the “survreg” function with a Weibull distribution (from the “survival” R package) and the “weibullReg” function (from the SurvRegCensCov’ R package).^7^, ^8^ Censored shedding times were stored as survival objects (e.g., 1+, 15+, or [1,6] days, as shown in Figure S3) using the survival package,^8^ which were then used in the AFT models. To compare viral load by prior immunity, we used Wilcoxon rank‐sum tests (serostatus), linear regression (titer), and plotted by age with loess smoother (ggplot's geom_smooth). All analyses were performed in R version 4.1.2.^9^ using the tidyverse,^10^ and code is available on GitHub (https://github.com/hannahma/SARS-CoV-2_shedding).

## 3 RESULTS

Three SARS‐CoV‐2 waves occurred in Managua, taking place roughly from May to July 2020, April–October 2021, and January–March 2022 (Figure 1A). The second wave was predominantly gamma and delta variants,^6^ and the third was presumably omicron.^11^ Across the three waves, 757 RT‐PCR‐confirmed SARS‐CoV‐2 infections were detected, 745 of which had prior ELISA data (Table S1 and Figure S4); 262 (35%) of infections were in index cases, and 483 (65%) were in household contacts. The full cohort of 2539 participants aged 0 to 94 years with an average household size of 5.1 people (range 2–12) has been described previously.^3^, ^6^ Sampling aFnd RT‐PCR results for each of the 745 infections are displayed in Figure S5. The age and sex distribution of participants with SARS‐CoV‐2 infections was similar to that of the full cohort—sex is balanced among children, but there is somewhat lower representation by adult males (Figure S6). Blood samples for ELISAs nicely book‐ended the first two waves and preceded the third wave (Figure 1; specific timing of screening and titer samples preceding infections are shown in Figure 1B,C, and their results are shown in Figure 1D,E). There were 145 infections having ≥1 vaccine dose ≥14 days prior, which were excluded from the primary analysis, and 53 infections among those fully vaccinated ≥14 days prior (Table S1 and Figure 1F).

### 3.1 Viral shedding duration and prior infection

**FIGURE 2:**
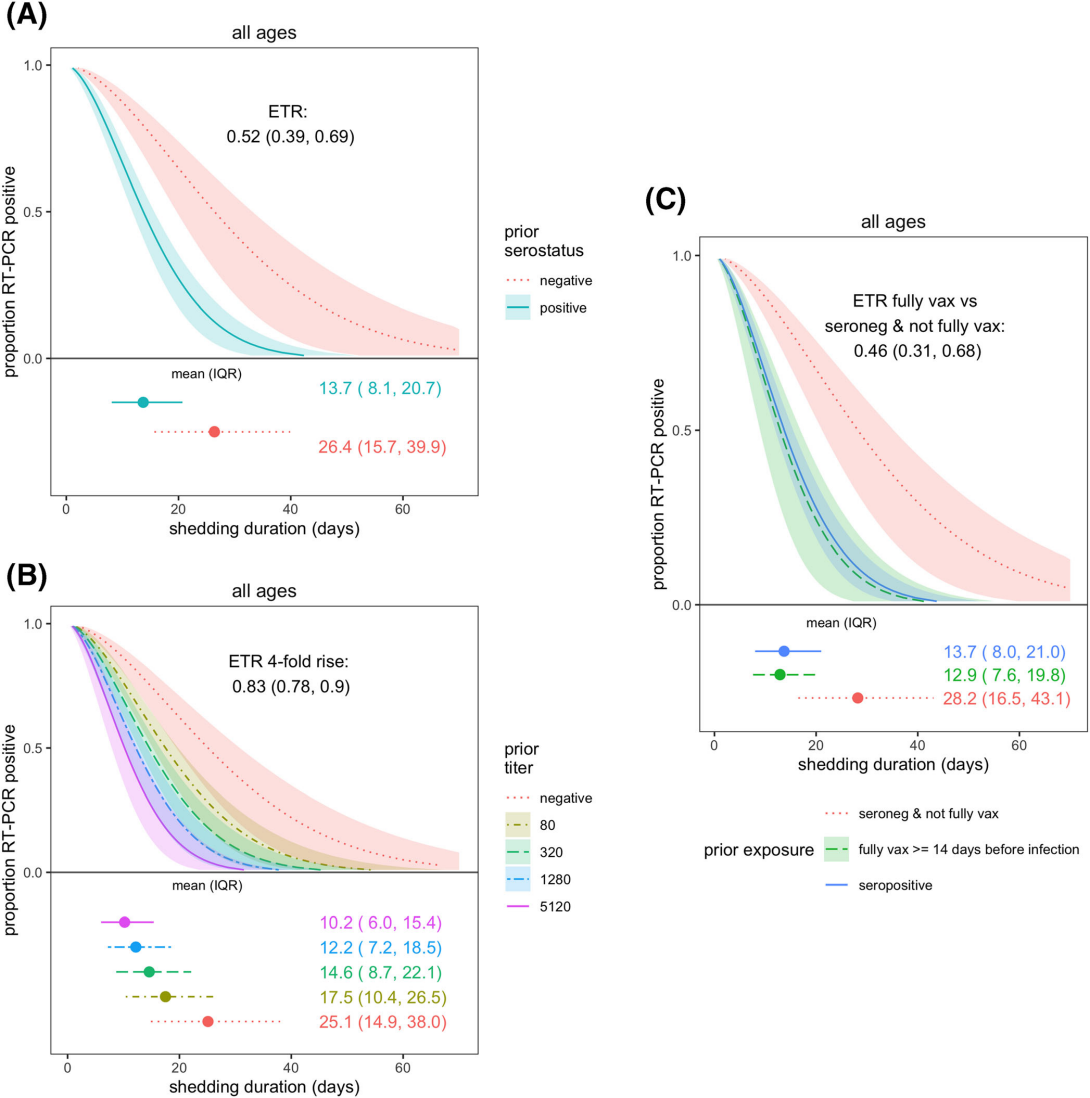
SARS‐CoV‐2 viral shedding duration by prior immunity. Prior immunity was measured as (A) serostatus and (B) anti‐spike titer. (C) Compares full vaccination and serostatus. Results are from accelerated failure time (AFT) models. Shaded regions represent 95% confidence intervals. Estimated mean and interquartile range (IQR) shedding durations are displayed graphically and in text below each figure. Individuals with ≥1 vaccination ≥14 days prior to infection were excluded from analyses in (A) and (B).

Using the AFT model, the mean shedding duration was 17.1 days (IQR: 9.91–26.3 days). Overall, there were no apparent differences in SARS‐CoV‐2 shedding duration by age group, sex, or obesity (Figure S7). Prior infection was associated with 48% shorter shedding (event time ratio [ETR] 0.52, 95% CI: 0.39–0.69; Figure 2A). The mean shedding for prior infected versus naïve individuals were 13.7 days (IQR: 8.1–20.7) versus 26.4 days (IQR: 15.7–39.9; Figure 2A). A fourfold higher spike titer was associated with 17% shorter shedding (ETR 0.83, 95% CI: 0.78–0.90; Figure 2B); those with the highest titers of 5120 shed on average 10.2 days (IQR: 6.0–15.4). Full vaccination was associated with a similar level of shortened shedding (ETR fully vaccinated vs. seronegative: 0.46, 95% CI: 0.31–0.68; Figure 2C).

**FIGURE 3:**
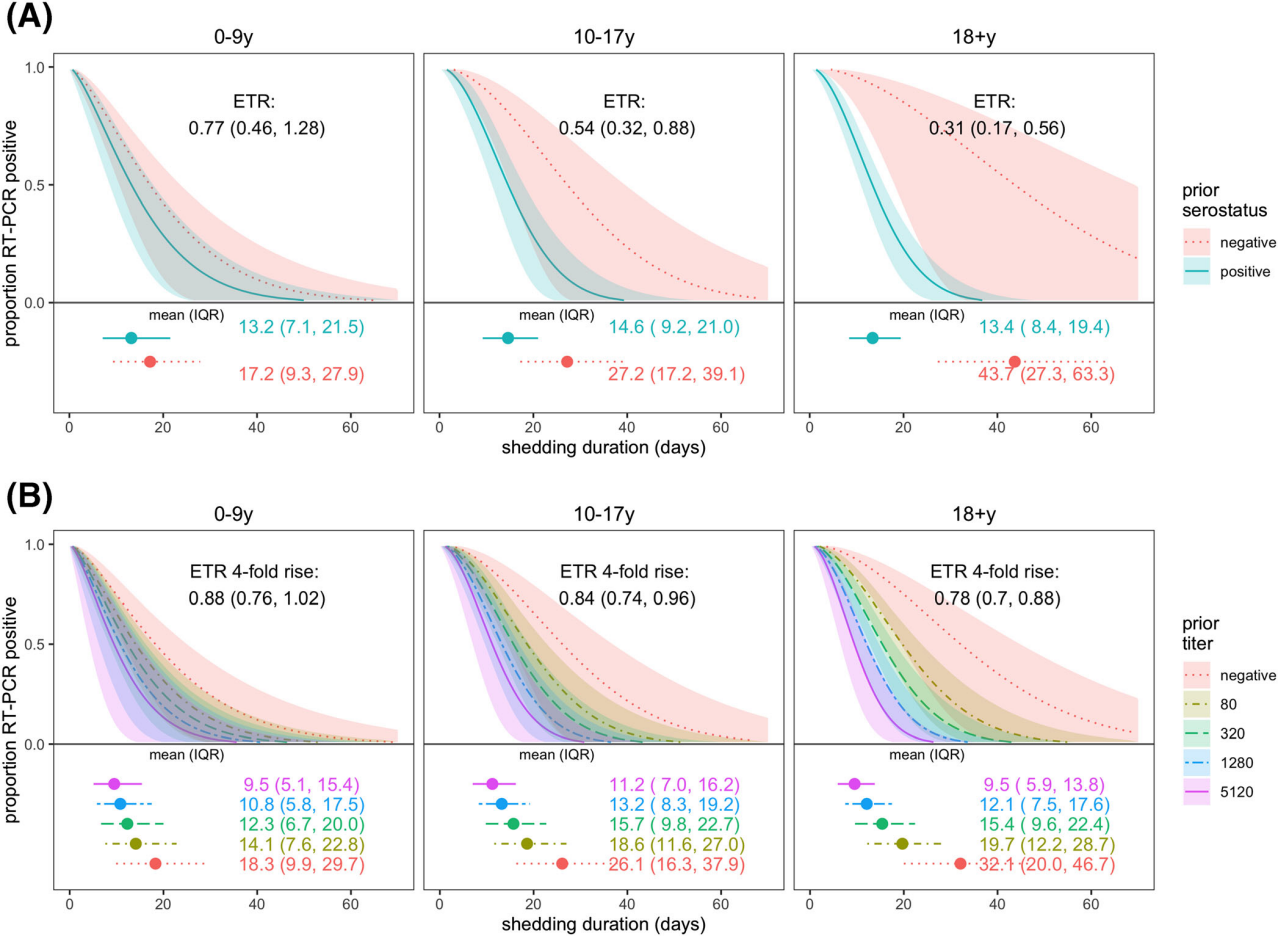
SARS‐CoV‐2 viral shedding duration by prior immunity and age. Prior immunity was measured as (A) serostatus and (B) anti‐spike titer. Results are from accelerated failure time (AFT) models. Shaded regions represent 95% confidence intervals. Estimated mean and interquartile range (IQR) shedding durations are displayed graphically and in text below each figure. Individuals with ≥1 vaccination ≥14 days prior to infection were excluded.

In adults and older children (10–17 years), prior infection was associated with shortened shedding (ETRs 0.31, 95% CI: 0.17–0.56 and 0.54, 95% CI: 0.32–0.88, respectively), but there was little difference for children aged 0–9 years by prior infection status (ETR 0.77, 95% CI: 0.46–1.28; Figure 3A). Similarly, fourfold higher titers in adults and older children were also associated with shorter shedding but not significantly associated for children aged 0–9 years (Figure 3B; ETRs for adults, older children, and younger children: 0.78, 95% CI: 0.70–0.88; 0.84, 95% CI: 0.74–0.96; and 0.88, 95% CI: 0.76–1.02, respectively).

When stratified by serostatus, naïve adults shed three times as long as naïve children aged 0–9 years (ETR 3.1, 95% CI: 1.48–6.52), but there was no difference in shedding times between prior infected children and adults (Figure S6A). Sex and obesity were not associated with shedding duration (Figure S7B,C).

### 3.2 Prior infection and viral load

**FIGURE 4:**
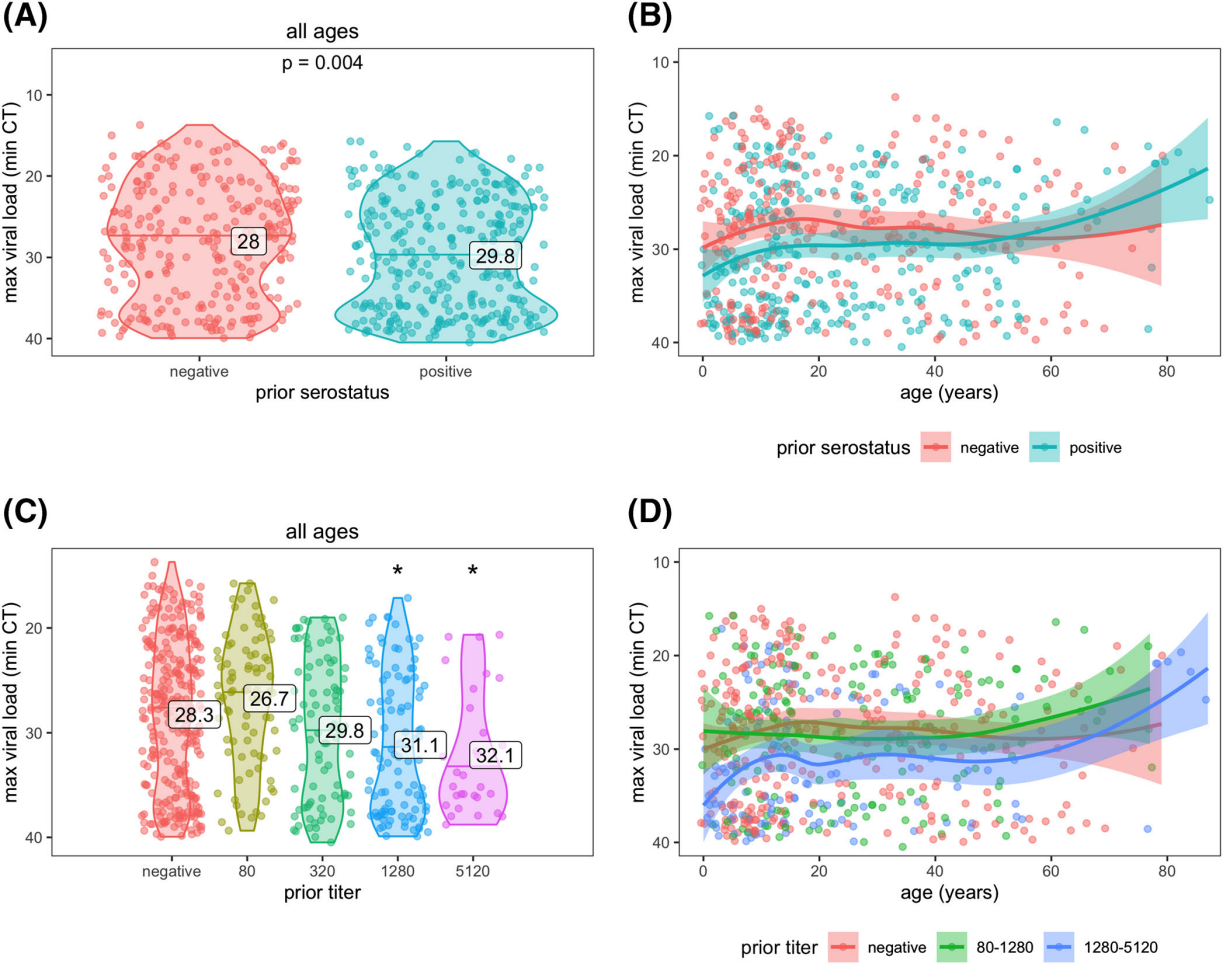
SARS‐CoV‐2 viral load by prior immunity and age. Prior immunity was measured as serostatus (A and B) and anti‐spike titer (C and D). Each data point represents an infection. Violin plots (A and C) show the distribution of viral load, by each level of immunity; horizontal lines indicate median CT values and printed numbers represent mean CT values. Asterisks in (C) indicate mean CT values significantly different from those in the negative group (*p* < 0.05). Line plots (B and D) are fitted with a loess smoother and shaded regions represent 95% confidence intervals.

Maximum viral loads, measured by RT‐PCR cycle threshold (CT; higher CT = lower viral load), were slightly lower for previously infected versus naïve individuals (mean 29.8 vs. 28.0 cycles, *p* = 0.0004; Figure 4A). Higher anti‐spike titers were also associated with a lower maximum viral load (32.1 vs. 28.3 cycles for titers of 5120 vs. negative; Figure 4C). Viral loads were somewhat lower across all ages for those with prior immunity (Figure 4B,D), except for participants aged above 60 years.

## 4 DISCUSSION

We found that prior infection‐induced immunity was associated with shorter shedding duration in adults and older children but not in younger children. Naïve adults shed longer than naïve children, but previously infected adults and children shed for similar durations. Although RT‐PCR is not a direct indicator of viral viability or infectiousness, RT‐PCR trajectories did roughly track with viable virus in a human challenge study.^12^ Given this, our finding of shorter durations of RT‐PCR‐detected viral shedding among prior infected may still translate to lower transmission from people who have been previously infected. These results suggest that as immunity is established, children may contribute proportionally more to transmission, and transmission will decrease (because of both lower susceptibility and lower transmissibility from shorter shedding times).

Our long shedding times are in line with what others have found—a meta‐analysis of 79 studies also found a mean shedding time of 17 days.^13^ Vaccination has been shown to shorten viral shedding.^14^ Lower viral loads (higher CT) have been found in reinfections (4.0 cycles) and vaccine breakthrough infections (1–3 cycles).^15^ We could not find any other studies comparing viral shedding duration by the level of infection‐induced prior immunity.

A limitation of our work is that blood samples were not available shortly before all infections—a few were from ~a year prior (Figure 1B,C). During long delays, (1) subsequent undetected infections could occur and (2) antibody titers may decline. Fortunately, the study design allows us to detect many even inapparent infections. And if the antibodies were lower than at the time of measurement, we would expect to see an even stronger association. Another limitation is that some of our observed infections were already positive on their first samples (right censored shedding durations), which also means that we may not have sampled at the highest viral loads; we would have more accurate estimates if we could observe the full shedding durations. We did not have a large enough sample size (with the censored data) to look at viral shedding in older adults, as we did with viral load. Because of epidemiological patterns, where everyone infected in the first wave was naïve and by the time 2022 began and Omicron was introduced, almost everyone in the cohort had been previously infected at least once, we were underpowered to examine the association of shedding duration with prior infection by year or variant.

In addition to vaccination, prior infection will have a major impact on the future of the SARS‐CoV‐2 pandemic and should be duly considered.

## CONFLICTS OF INTEREST

Aubree Gordon serves on an advisory board for Janssen and has received consulting fees from Gilead Sciences. All other authors report no competing interests.

## AUTHOR CONTRIBUTIONS

Conceptualization: Aubree Gordon. Data curation: Hannah E. Maier. Formal analysis: Hannah E. Maier. Funding Acquisition: Aubree Gordon. Investigation: Angel Balmaseda, Carlos Barilla, Guillermina Kuan, Roger Lopez, Sergio Ojeda Miguel Plazaola, Nery Sanchez, Saira Saborio. Methodology: Angel Balmaseda, Aubree Gordon, Guillermina Kuan, Hannah E. Maier. Project administration: Angel Balmaseda, Aubree Gordon, Sergio Ojeda, Guillermina Kuan. Resources: Aubree Gordon. Software: Hannah E. Maier. Supervision: Angel Balmaseda, Aubree Gordon, Guillermina Kuan. Visualization: Hannah E. Maier. Writing—original draft: Hannah E. Maier. Writing—review and editing: Angel Balmaseda, Carlos Barilla, Aubree Gordon, Guillermina Kaun, Roger Lopez, Hannah E. Maier, Sergio Ojeda, Miguel Plazaola, Saira Saborio, Nery Sanchez.

## ETHICS AND CONSENT

This study was approved by the institutional review boards at the Nicaraguan Ministry of Health and the University of Michigan (HUM00119145 and HUM00178355). Informed consent or parental permission was obtained for all participants. Assent was obtained from children aged ≥6 years.

### PEER REVIEW

The peer review history for this article is available at https://publons.com/publon/10.1111/irv.13074.

## Supporting information


**Table S1.** Characteristics of SARS‐CoV‐2 infections
**Figure S1.** Cohort study diagram for HICS
**Figure S2.** Nested transmission study diagram for SARS‐CoV‐2 activations in HICS
**Figure S3.** Example of shedding time censoring
**Figure S4.** Study flowchart
**Figure S5.** Sampling and RT‐PCR results for all 745 infections
**Figure S6.** Population pyramid for SARS‐CoV‐2 infections
**Figure S7.** SARS‐CoV‐2 viral shedding duration by age, sex, and obesity among prior seronegative and seropositive

## Data Availability

De‐identified data needed to create the figures and R code is available on GitHub (https://github.com/hannahma/SARS-CoV-2_shedding). As this is a human subjects study, the full data are not publicly available. However, individual‐level data may be shared with outside investigators who submit a proposal for review by the study executive committee following University of Michigan and Nicaraguan IRB approval.
