# Supplementary material for: SARS‐CoV‐2 infection‐induced immunity and the duration of viral shedding: Results from a Nicaraguan household cohort study
Source: Influenza Other Respir Viruses. 2022 Dec 1;17(1):e13074. doi: 10.1111/irv.13074 (PMC9835439; doi:10.1111/irv.13074)
Supplement: Supplementary file 1 — Table S1. Characteristics of SARS‐CoV‐2 infections Figure S1. Cohort study diagram for HICS Figure S2. Nested transmission study diagram for SARS‐CoV‐2 activations in HICS Figure S3. Example of shedding time censoring Figure S4. Study flowchart Figure S5. Sampling and RT‐PCR results for all 745 infections Figure S6. Population pyramid for SARS‐CoV‐2 infections Figure S7. SARS‐CoV‐2 viral shedding duration by age, sex, and obesity among prior seronegative and seropositive [file IRV-17-0-s001.docx]

Supplement for

**SARS-CoV-2 infection-induced immunity and the duration of viral shedding: results from a Nicaraguan household cohort study**

Hannah E. Maier, Ph.D. ^1^, Miguel Plazaola, M.D ^2^, Roger Lopez, MPH^2,3^, Nery Sanchez, M.D.^2^, Saira Saborio, MS^2,3^, Sergio Ojeda, M.D.^2,4^, Carlos Barilla, BA^2^, Guillermina Kuan, M.D.^2,4^, Angel Balmaseda, M.D. ^2,3^, Aubree Gordon, Ph.D.^1*^

^1^Department of Epidemiology, School of Public Health, University of Michigan in Ann Arbor, Michigan, USA, ^2^Sustainable Sciences Institute, Managua, Nicaragua, ^3^Centro Nacional de Diagnóstico y Referencia at the Ministry of Health, Managua, Nicaragua, ^4^Centro de Salud Sócrates Flores Vivas at the Ministry of Health, Managua, Nicaragua,

* corresponding author email: [gordonal@umich.edu](mailto:gordonal@umich.edu) (A.G.)

Table of Contents

Table S1 - Characteristics of SARS-CoV-2 infections

Fig S1 - Cohort study diagram for HICS

Fig S2 - Nested transmission study diagram for SARS-CoV-2 activations in HICS

Fig S3 - Example of shedding time censoring

Fig S4. Study flowchart

Fig S5 - Sampling and RT-PCR results for all 745 infections

Fig S6. Population pyramid for SARS-CoV-2 infections

Fig S7 - SARS-CoV-2 viral shedding duration by age, sex, and obesity among prior seronegative and seropositive

Table S1. Characteristics of SARS-CoV-2 infections

|  | Previously Seronegative  (N=302, 40.5%) | Previously Seropositive  (N=443, 59.5%) | Total Infections  with prior ELISA data  (N=745, 100%) |
| --- | --- | --- | --- |
| Age |  |  |  |
| 0-4y | 89 (29.5%) | 88 (19.9%) | 177 (23.8%) |
| 5-17y | 67 (22.2%) | 112 (25.3%) | 179 (24.0%) |
| 18+y | 146 (48.3%) | 243 (54.9%) | 389 (52.2%) |
| Sex |  |  |  |
| F | 183 (60.6%) | 280 (63.2%) | 463 (62.1%) |
| M | 119 (39.4%) | 163 (36.8%) | 282 (37.9%) |
| At least 1 vaccine dose ≥14 days before infection | 24 (7.9%) | 121 (27.3%) | 145 (19.5%) |
| Fully vaccinated ≥14 days before infection | 4 (1.3%) | 49 (11.1%) | 53 (7.1%) |


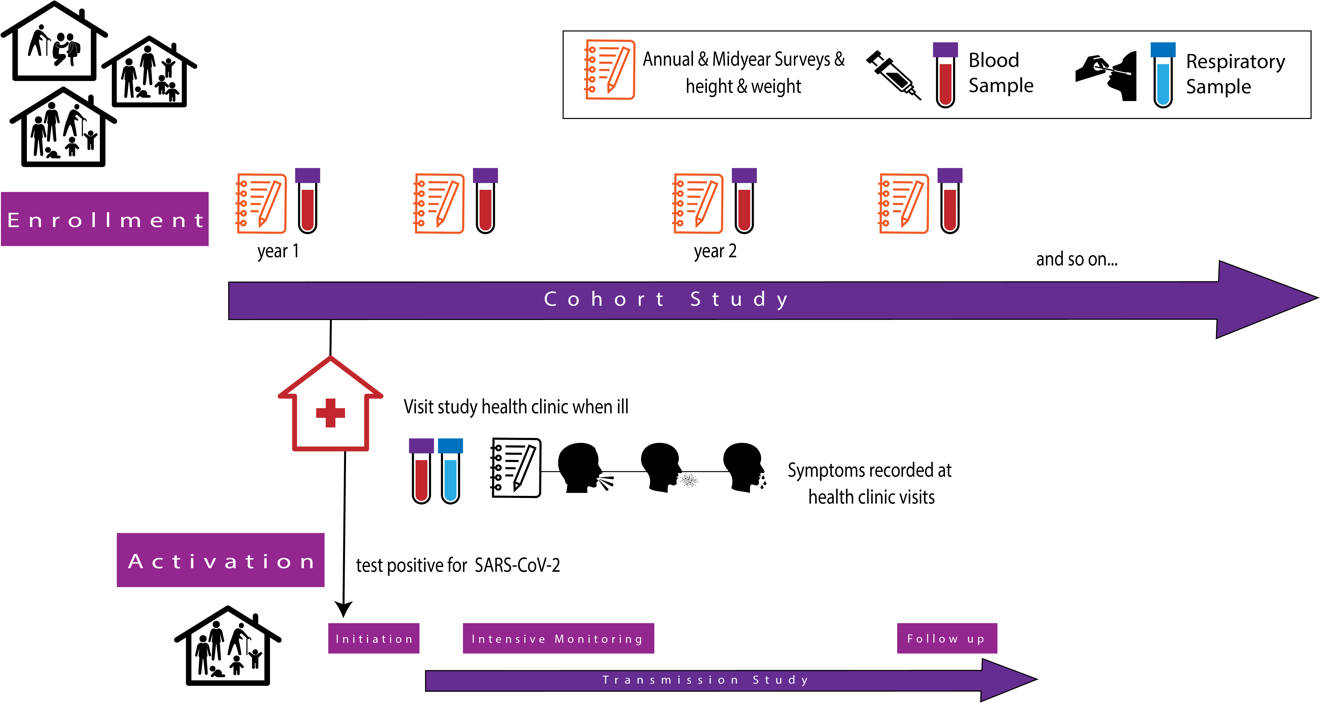


Fig S1. Cohort study diagram for HICS.


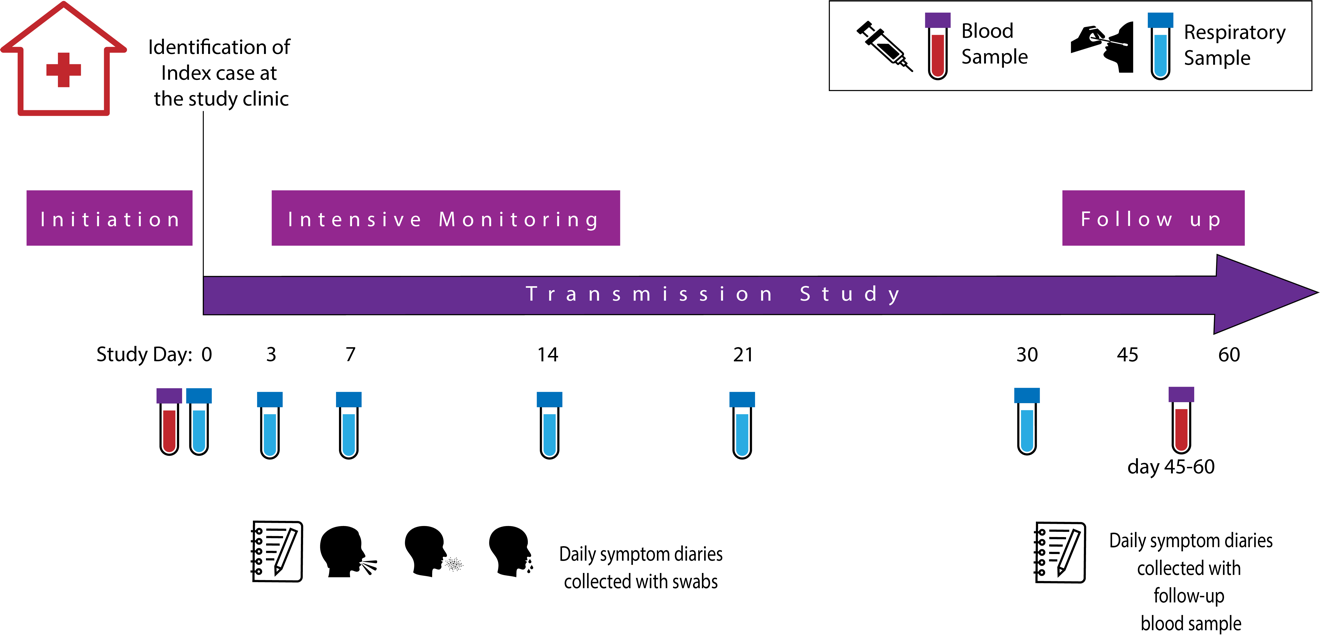


Fig S2. Nested transmission study diagram for SARS-CoV-2 activations in HICS.


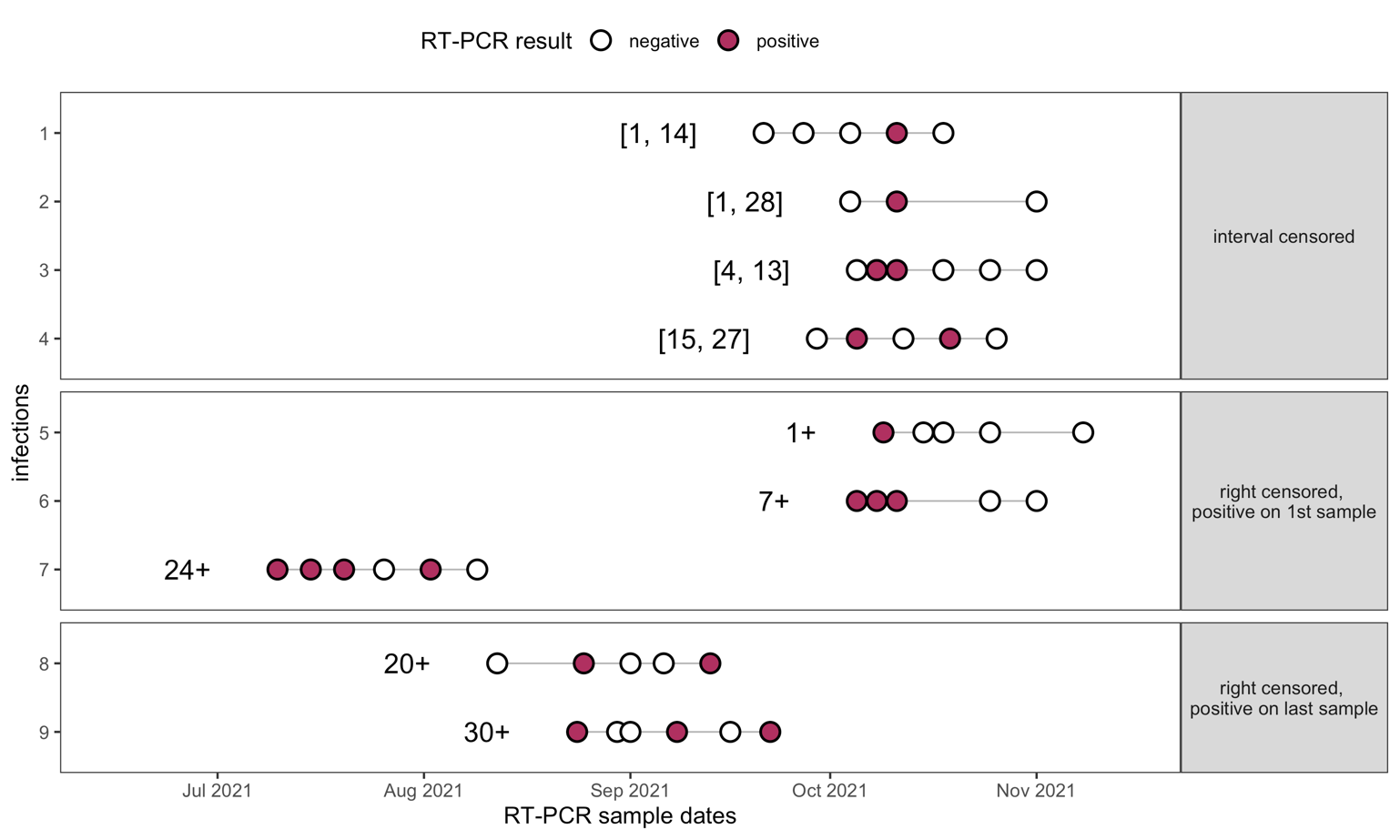


Fig S3. Example of shedding time censoring. Shedding times listed are formatted as survival objects, created with the ‘survival’ package.


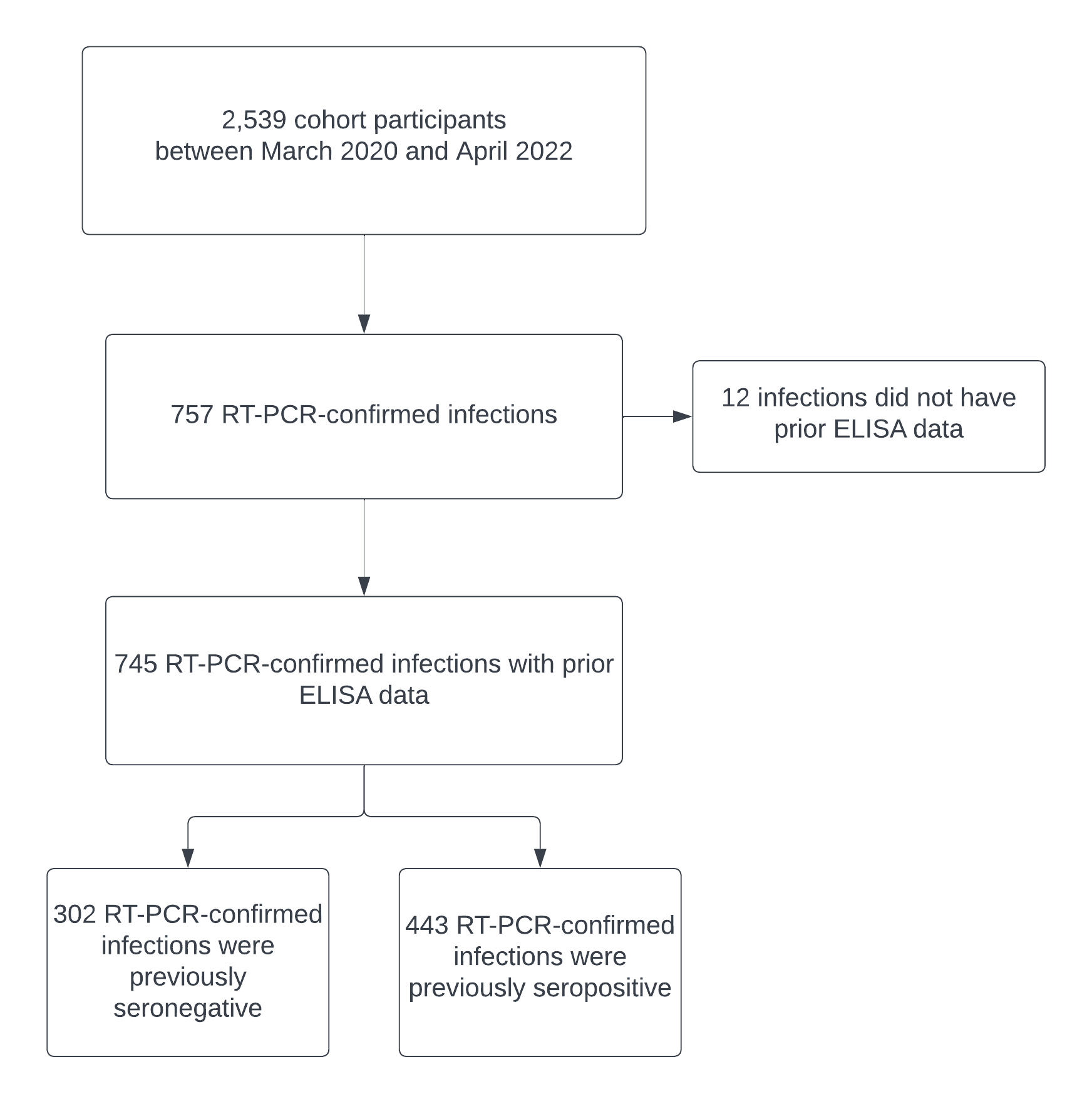


Fig S4. Study flowchart.


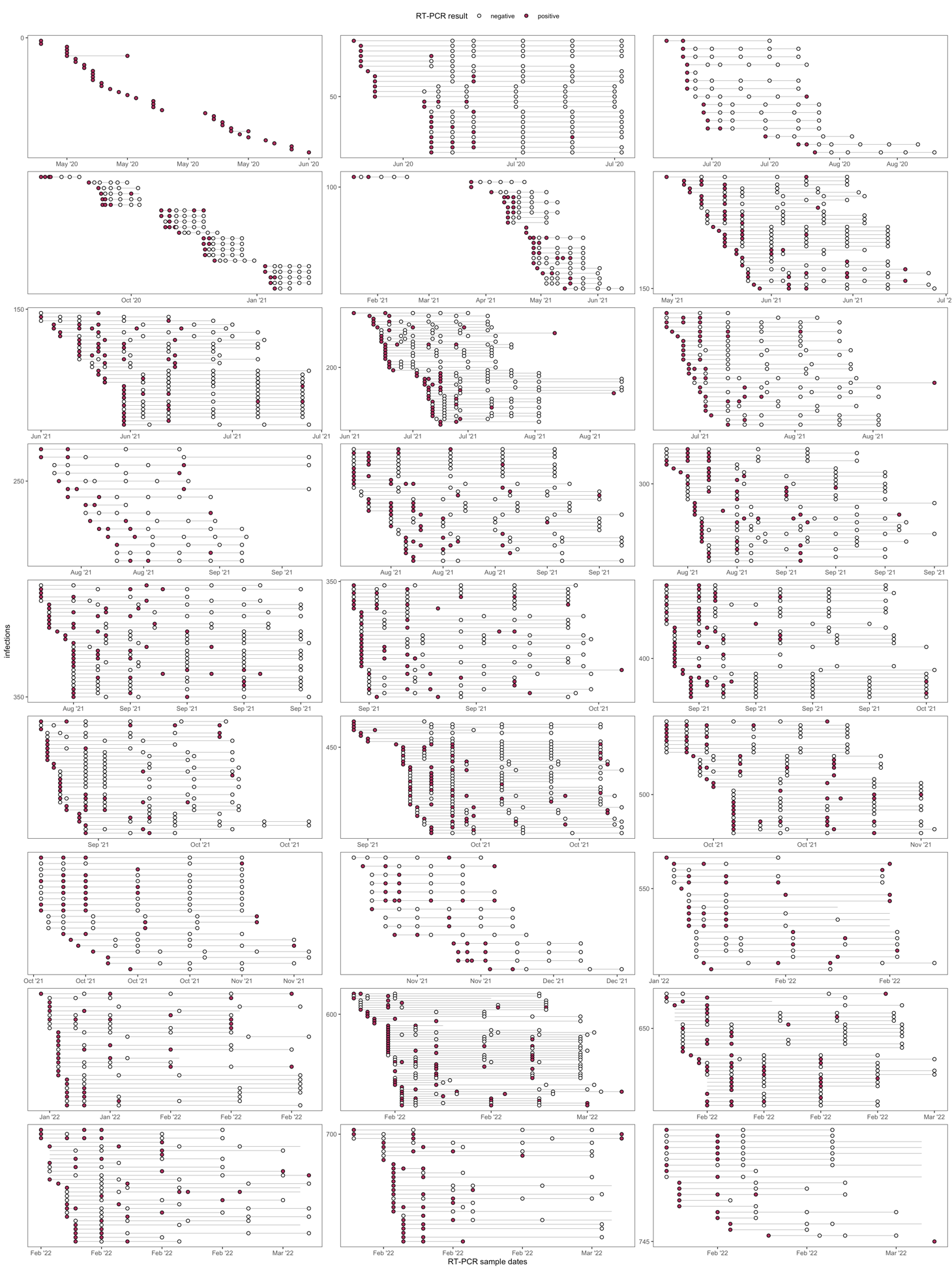


Fig S5. Sampling and RT-PCR results for all 745 infections.

*
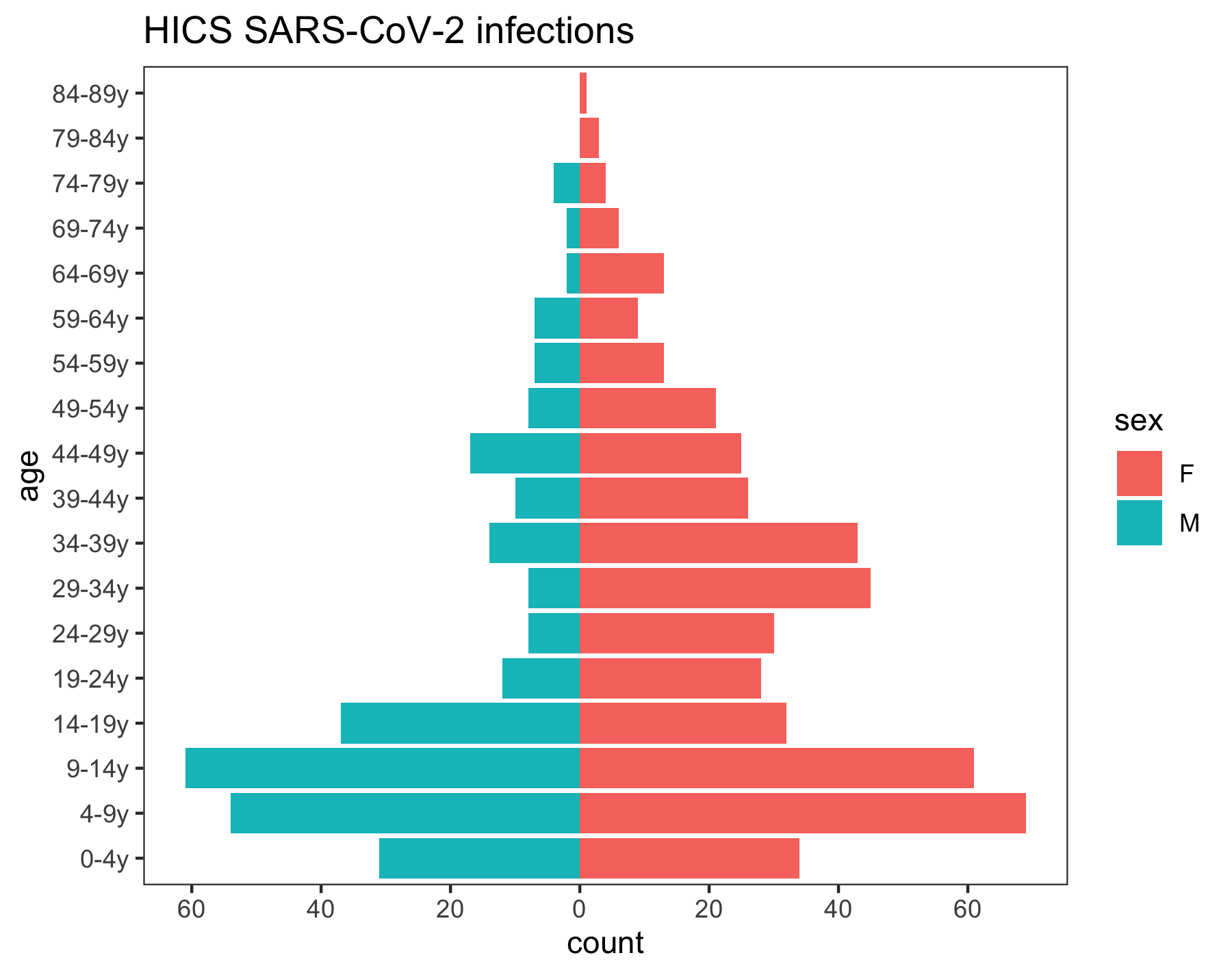
*

Figure 6. Population pyramid for SARS-CoV-2 infections.


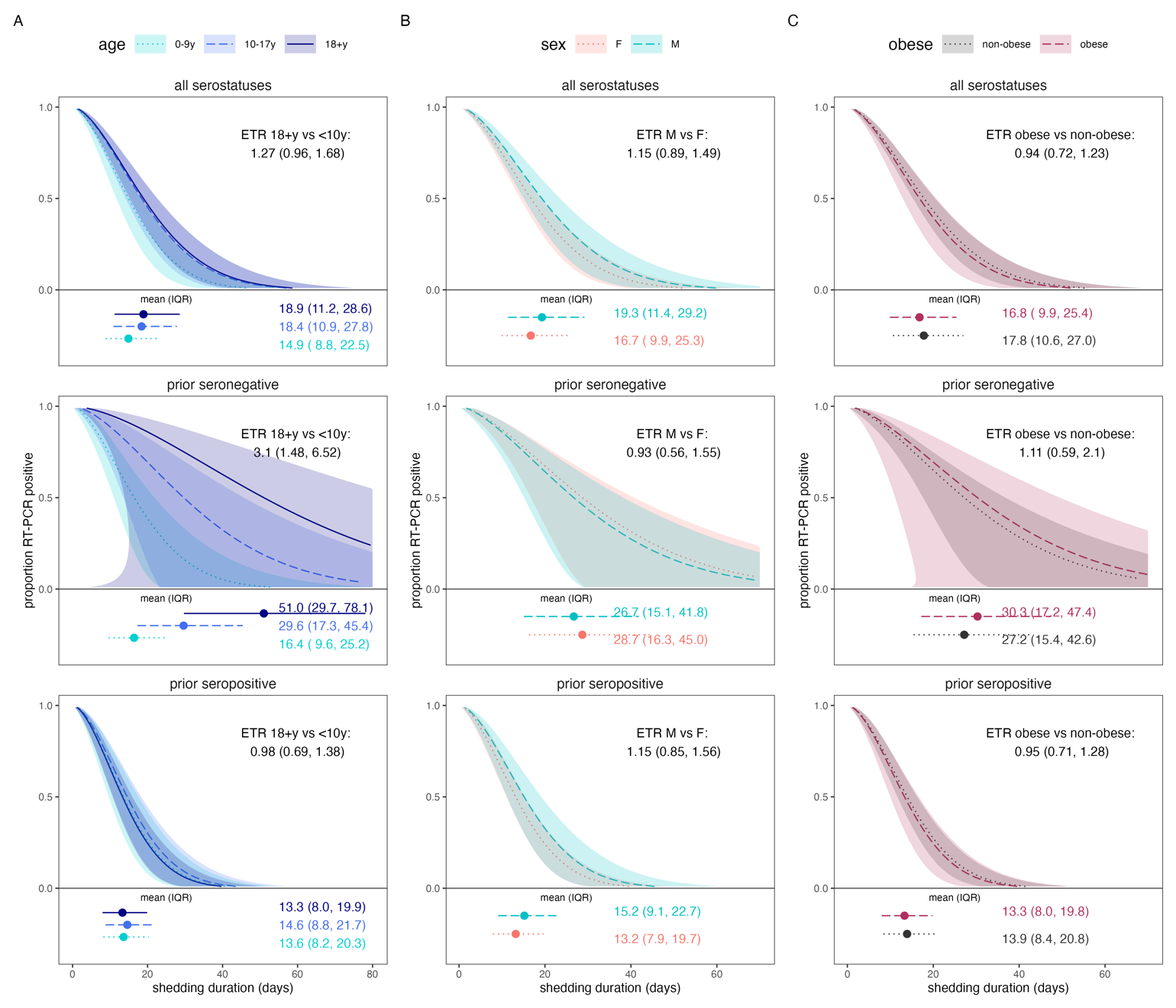


Fig S7. SARS-CoV-2 viral shedding duration by age, sex, and obesity among prior seronegative and seropositive. Results are from accelerated failure time (AFT) models. Shaded regions represent 95% confidence intervals. Estimated mean and interquartile range (IQR) shedding durations are displayed graphically and in text below each figure. Individuals with ≥1 vaccination ≥14 days prior to infection were excluded.
